# Comparative Genomic and Metagenomic Investigations of the Corynebacterium tuberculostearicum Species Complex Reveals Potential Mechanisms Underlying Associations To Skin Health and Disease

**DOI:** 10.1128/spectrum.03578-22

**Published:** 2022-12-21

**Authors:** Rauf Salamzade, Mary Hannah Swaney, Lindsay R. Kalan

**Affiliations:** a Department of Medical Microbiology and Immunology, School of Medicine and Public Health, University of Wisconsin, Madison, Wisconsin, USA; b Microbiology Doctoral Training Program, University of Wisconsin, Madison, Wisconsin, USA; c Department of Medicine, Division of Infectious Disease, School of Medicine and Public Health, University of Wisconsin, Madison, Wisconsin, USA; d M.G. DeGroote Institute for Infectious Disease Research, David Braley Centre for Antibiotic Discovery, Department of Biochemistry and Biomedical Sciences, McMaster University, Hamilton, Ontario, Canada; Lerner Research Institute

**Keywords:** *Corynebacterium*, atopic dermatitis, metagenomics, skin microbiome, species complex, taxonomy, tuberculostearicum

## Abstract

*Corynebacterium* are a diverse genus and dominant member of the human skin microbiome. Recently, we reported that the most prevalent *Corynebacterium* species found on skin, including Corynebacterium tuberculostearicum and *Corynebacterium kefirresidentii*, comprise a narrow species complex despite the diversity of the genus. Here, we apply high-resolution phylogenomics and comparative genomics to describe the structure of the *C. tuberculostearicum* species complex and highlight genetic traits which are enriched or depleted in it relative to other *Corynebacterium*. Through metagenomic investigations, we also find that individual species within the complex can associate with specific body sites. Finally, we discover that one species from the complex, *C. kefirresidentii*, increases in relative abundance during atopic dermatitis flares, and show that most genomes of this species encode a colocalized set of putative virulence genes.

**IMPORTANCE** *Corynebacterium* are commonly found bacteria on the human skin. In this study, we perform comparative genomics to gain insight into genetic traits which differentiate a phylogenetically related group of *Corynebacterium*, the Corynebacterium tuberculostearicum species complex, that includes the most prevalent species from the genus in skin microbiomes. After resolving the presence of distinct species within the complex, we applied metagenomic analysis to uncover biogeographic associations of individual species within the complex with specific body sites and discovered that one species, commonly found in the nares of individuals, increases in abundance across multiple body sites during atopic dermatitis flares.

## INTRODUCTION

Several species of *Corynebacterium* have been isolated from human skin, often associated with diseases such as diphtheria and regarded as opportunistic pathogens (1, 2). Only recently, with the application of metagenomics to comprehensively survey the skin microbiome, have we begun to develop a better understanding of this understudied genus and assess mechanisms underlying their basis as core constituents of skin microbiomes (3–5).

Regarded as a lipophilic and disease-associated species, *C. tuberculostearicum* was first named and associated with cases of diphtheria in 1984 (1), later it was found to be associated with rhinosinusitis (6), and more recently it has been shown to elicit specific inflammatory signaling pathways of skin cells (5). While it was noted that *C. tuberculostearicum* could be closely related to other *Corynebacterium* species when it was first validated as a species in 2004 (7), this analysis was based on 16S rRNA sequences which provide limited resolution for such investigations. Later work has suggested that misclassification of *C. tuberculostearicum* is likely prevalent by clinical biochemical assays and that the species is potentially underreported (8).

We recently found that the three most prevalent species of *Corynebacterium* in healthy skin microbiomes from a metagenomic survey in our lab (9), currently classified as Corynebacterium tuberculostearicum, *Corynebacterium kefirresidentii*, and Corynebacterium aurimucosum type E in GTDB (10), belong to a narrow clade, which includes at least one additional species (11). Because the average nucleotide identity (ANI) between genomes in this clade is relatively high (>88%), we refer to the clade as the *C. tuberculostearicum* species complex (10, 11). While *C. aurimucosum* and *C. tuberculostearicum* have been validated as species (7, 12), reassessment of genomes classified as these species in NCBI’s GenBank database by GTDB suggests that some genomes are currently mislabeled in GenBank (10). Further, there currently exists three different *C. aurimucosum* type species in GTDB and, of these, only type E was found to belong to the *C. tuberculostearicum* species complex. *C. kefirresidentii* was only recently proposed as a new species and has not yet been validated by the International Committee on Systematics of Prokaryotes (13). For simplicity, in this study, we use the species names of *C. kefirresidentii* and *C. aurmicusoum* type E, in accordance with species labels in GTDB release 207.

Recently, a *C. kefirresidentii* isolate sampled from healthy human skin was found to be lipophilic, similar to *C. tuberculostearicum* (9). It was also shown to grow better in the presence of compounds commonly found in sweat (9). This finding is in accordance with our earlier observation that members of the *C. tuberculostearicum* species complex are commonly found at body sites regarded as moist (11). In this study, we apply comparative genomics and metagenomics analytics to develop a better understanding of the genetic factors underlying the prevalence of the *C. tuberculostearicum* species complex on healthy skin. In addition, we assess the biogeographical distribution of individual species within the complex across different skin body sites and skin conditions.

## RESULTS

### A high-resolution phylogeny of the *C. tuberculostearicum* species complex.

A set of 26 genomes belonging to the *C. tuberculostearicum* species complex was used for phylogenomic investigation of the species complex (Table S1). This set of genomes includes 22 genomes we had previously described as part of this complex (11). In addition, we added three recently reported metagenome-assembled genomes (MAGs) from skin predicted to correspond to novel *Corynebacterium* species (14) and the genome of a *C. kefirresidentii* we recently isolated from human skin (9). All 26 genomes, including a total of eight MAGs, were regarded as high completion and showed low rates of contamination by CheckM analysis (15) (Table S1).

**FIG 1:**
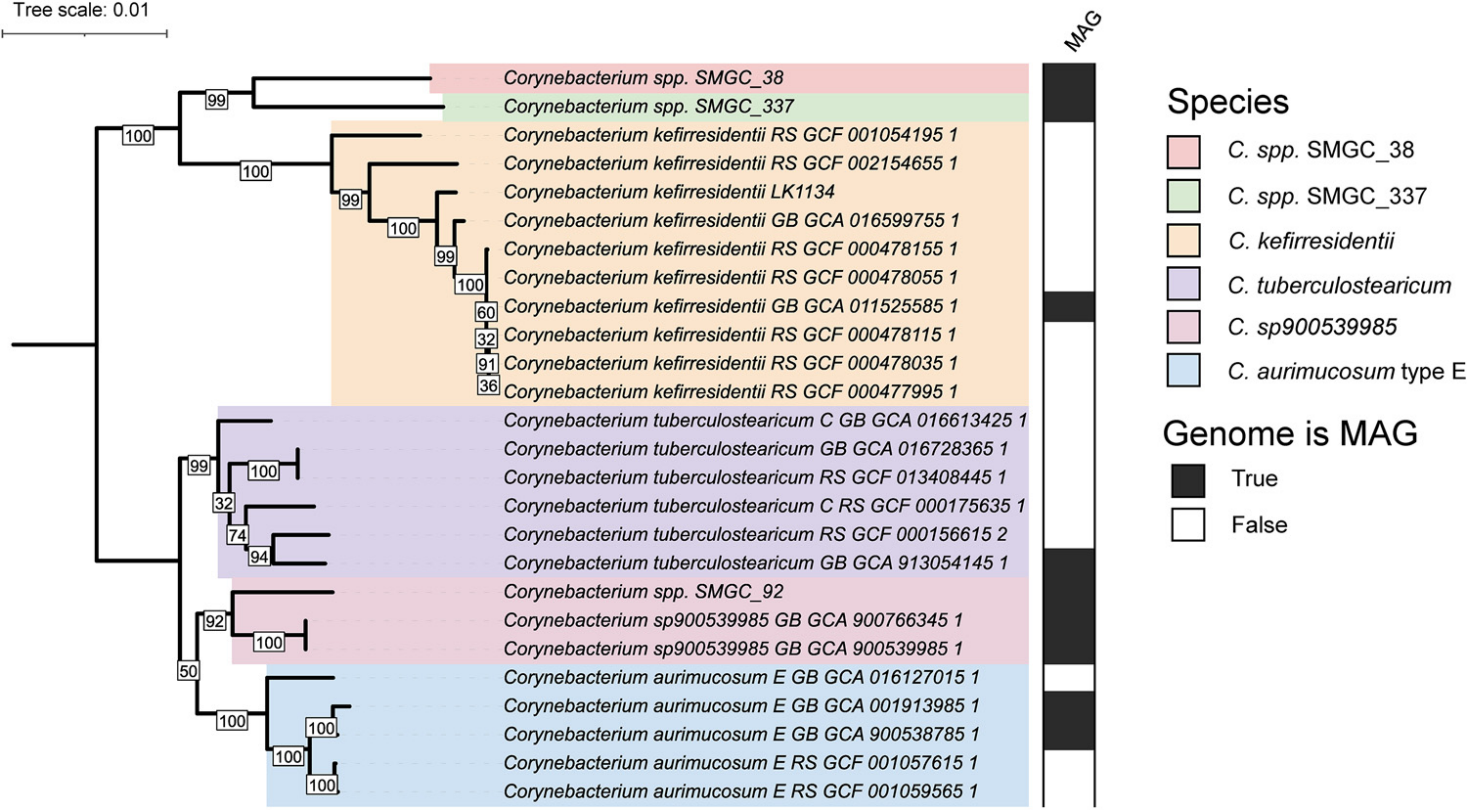
A high-resolution phylogenomic view of the *C. tuberculostearicum* species complex. A maximum-likelihood phylogeny of the *C. tuberculostearicum* species complex, constructed from 15,936 sites predicted to not be impacted by recombination and not exhibit ambiguity in any of the 26 genomes, is shown after midpoint rooting. Bootstrap values are indicated at inner nodes.

A set of 1,250 single-copy-core (SCC) orthologs was identified among the 26 genomes belonging to the species complex and used to construct a high-resolution phylogeny following filtering of sites predicted to be affected by recent or ancestral recombination (16, 17) (Fig. 1). This phylogeny was found to be highly concordant with species classifications for the genomes by GTDB-tk (18) run with GTDB release 207 (10), but not their taxonomic designations on GenBank (Table S1).

ANI between genomes belonging to different species ranged from a lower limit of ~88% (11) to nearly 95% (Fig. S1A), while 99.4% of pairwise comparisons between genomes from the same species had an ANI of 95% or greater. Within the subclade containing the species *Corynebacterium sp900539985*, *C. aurimucosum* type E, and *C. tuberculostearicum*, genomes from different species were found to exhibit high ANI values, often exceeding 94%, which is close to the 95% threshold commonly used to delineate species with the metric (19, 20) and suggests recent speciation. Similarly, the ANI between genomes *C.* spp. SMGC_38 and *C.* spp. SMGC_337 was estimated as being between 93% and 95%. These two newly reported species (14) exhibited greater than 90% ANI to *C. kefirresidentii* genomes (Fig. S1A). The MAG proposed to correspond to a novel *Corynebacterium* species (14), *Corynebacterium* spp. SMGC_92, was found to exhibit high ANI to an established but currently unnamed species in GTDB, *C.* sp900539985, and designated as belonging to this species. Due to close phylogenetic placement and ANI estimates exceeding 95%, we further propose that GTDB species *C. tuberculostearicum* and *C. tuberculostearicum* type C should simply be regarded as one species (*C. tuberculostearicum*). In addition, genomes classified as *C. aurimucosum* type E were significantly different than genomes classified as *C. aurimucosum* or *C. aurimucosum* type C in GTDB, which lie outside the *C. tuberculostearicum* species complex (Fig. S1B).

Overall, the phylogeny constructed using the core-genome with sites predicted to be affected by recombination removed, was well supported as indicated by high bootstrap values at ancestral nodes to species and sets of species (Fig. 1). However, we were unable to confidently resolve whether *C. sp900539985* is more related to *C. tuberculostearicum*, as ANI analysis suggests, or type E *C. aurimucosum* (Fig. 1; Fig. S1A).

### Biogeographic distribution and identification of unique genes in the *C. tuberculostearicum* species complex.

**FIG 2:**
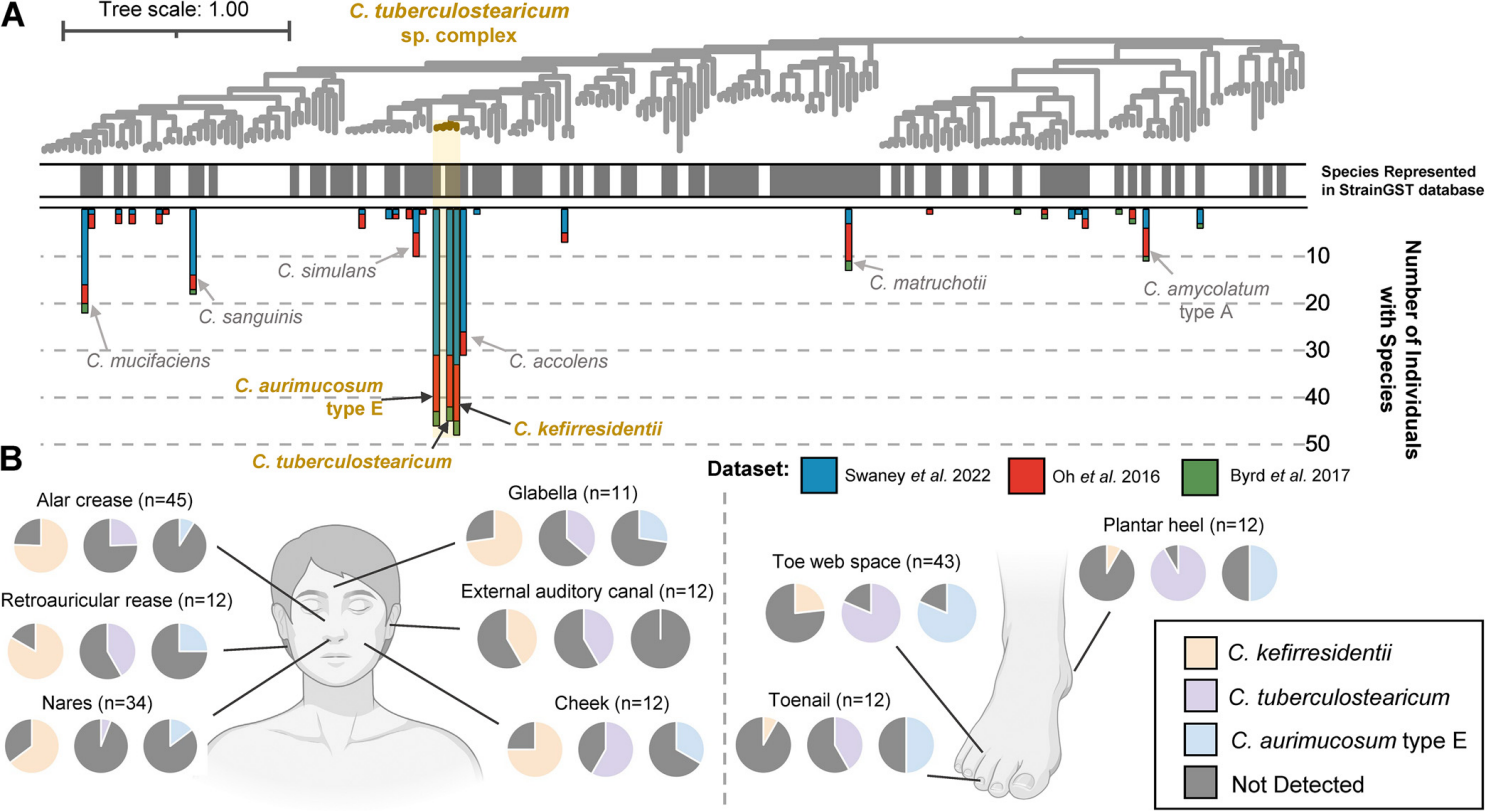
The *C. tuberculostearicum* species complex across skin metagenomic data sets and body sites. (A) A phylogeny of 187 distinct *Corynebacterium* representative genomes is shown with the *C. tuberculostearicum* species complex highlighted in gold. The color strip indicates species which were represented in the StrainGST database that we used to detect strains/species within metagenomes (gray). Bars correspond to the number of individuals from three skin-related metagenomic studies which were found to feature a species, with coloring representing the study. Only metagenomes corresponding to healthy controls from the Byrd et al. (22) study are considered. (B) The proportion of subjects from specific body sites of healthy adults with *C. kefirresidentii*, *C. tuberculostearicum*, and *C. aurimucosum* type E detected is shown based on metagenomes from the Oh et al. (3) and Swaney et al. (4) studies. The total number of subjects assessed for each body site is indicated in parentheses. BioRender was used for generating anatomical illustrations.

To address potential biases in reporting on the prevalence of the *C. tuberculostearicum* species complex found within skin metagenomes produced in our laboratory (4), we used StrainGST (21) to profile the presence of representative *Corynebacterium* genomes among additional independently published metagenomic data sets profiling the healthy skin microbiome (3), including healthy controls from a study focused on profiling the skin microbiome in atopic dermatitis, a common pediatric allergic disease (22). In concordance with our previous findings (11), we observed that all three species from the complex represented in the StrainGST database are highly prevalent in these additional metagenomic data sets relative to other *Corynebacterium* species (Fig. 2A; Table S2). We additionally tested and confirmed that StrainGST and our constructed database were highly reliable for detecting diverse strains lacking representation in the database using simulated read sets generated from public genomes (95.8% accuracy; Table S3). StrainGST was also highly accurate for detecting species presence in simulated mock metagenomic read sets consisting of simulated reads from five *Corynebacterium* strains (90% accuracy).

We additionally find that *C. tuberculostearicum* and *C. kefirresidentii* were differentially prevalent at distinct body sites. While *C. tuberculostearicum* was prevalent and found in high abundances at foot associated body sites, in particular the toe web space, *C. kefirresidentii* was more prevalent in nasal and surrounding body sites on the face (Fig. 2B; Fig. S3).

To understand the basis of the enrichment of the *C. tuberculostearicum* species complex on skin, we performed comparative genomics to determine if specific traits were found more commonly or less commonly than expected within the complex relative to the rest of the genus (Table S4). A representative set of 187 *Corynebacterium* genomes was selected based on dereplication (23) of those designated as belonging to the genus in GTDB at 95% ANI, to approximate selection of a single representative genome per species.

We identified only two homolog groups which were statistically significant for being absent in the species complex but highly conserved throughout the genus. These were predicted to encode a peptidase and a short-chain type oxidoreductase, respectively (Fig. S3A and B).

A larger set of proteins were identified as being statistically enriched in the *C. tuberculostearicum* species complex in relation to other *Corynebacterium*. We filtered for 26 homolog groups which were found to be nearly core to the species complex (found in ≥95% of the 26 *C. tuberculostearicum* genomes). While most of these homolog groups lacked reliable functional annotations, three were putatively identified and predicted to function as acetyltransferases. The largest homolog group, found in only 3.8% of *Corynebacterium* outside the species complex, was predicted to encode a collagen-like peptide (Fig. S3C and D) containing a standard signal peptide sequence, which suggested secretion by the Sec translocon (24). Only 0.01% of *Corynebacterium* proteins featured a collagen-like Gly-X-Y repeating motif of length 20 or greater, of which 18.6% belonged to this homolog group.

### *C. kefirresidentii* increases in relative abundance during atopic dermatitis flares.

**FIG 3:**
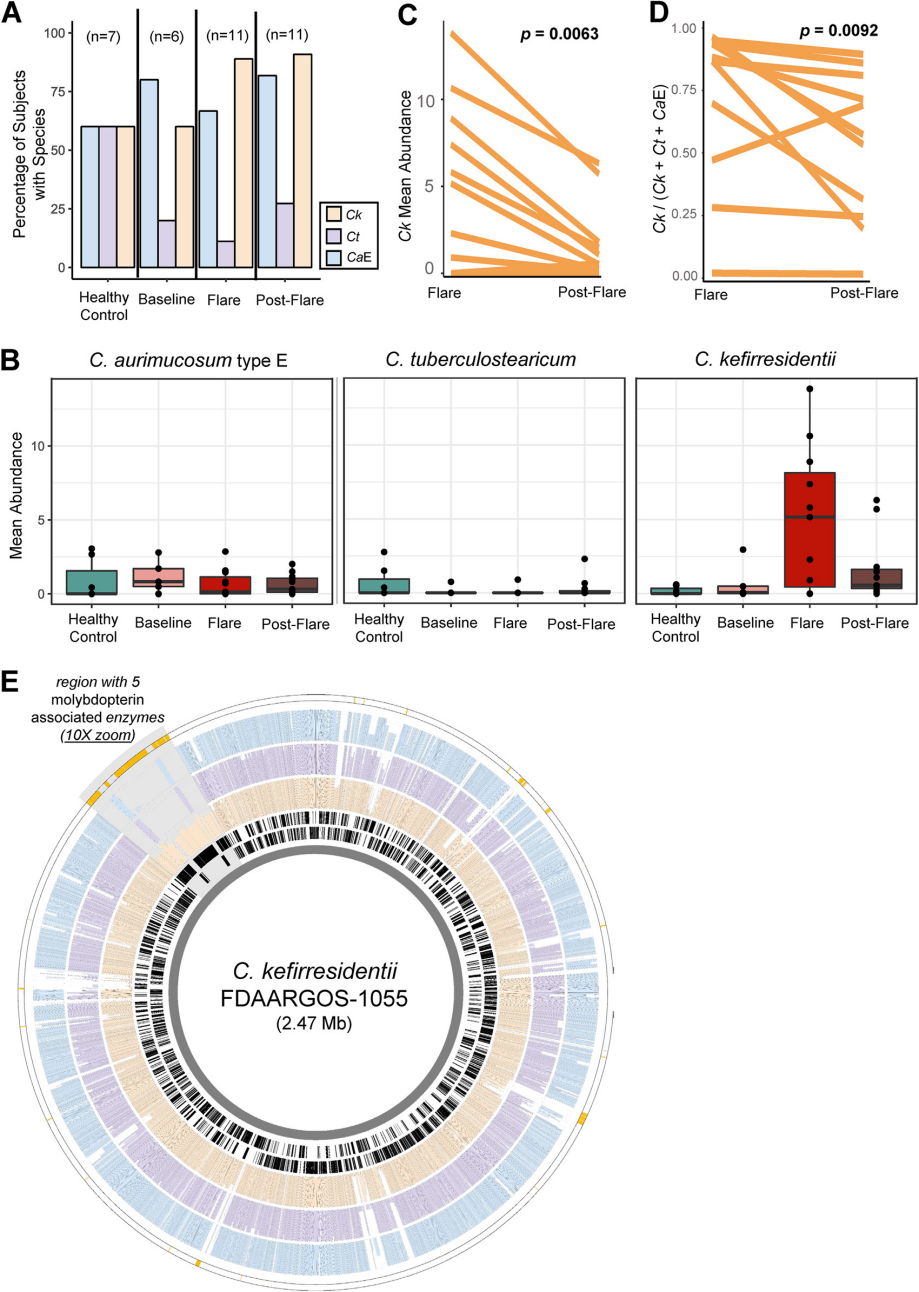
Increase in *C. kefirresidentii* relative abundance in metagenomes sampled during atopic dermatitis flares. (A) The percentage and (B) abundance of subjects featuring *C. kefirresidentii* (*Ck*), *C. tuberculostearicum* (*Ct*), and *C. aurimucosum* type E (*Ca*E) is shown for each AD disease state as well as healthy controls. Species abundance was calculated from averaging abundance of individual metagenomes across body-sites for each subject from the Byrd et al. (22) data set. (C) Changes in the StrainGST estimated relative abundance of *Ck* and (D) a statistic which measures the relative abundance of *Ck* to *Ct* and *Ca*E based on read alignment and coverage measurement of species-specific alleles along the core genome of the species complex is shown between paired flare and postflare surveys. (E) Genomic location of homolog groups identified as enriched in *C. kefirresidentii* genomes relative to *C. tuberculostearicum* and *C. aurimucosum* type E genomes along the chromosome of *C. kefirresidentii* FDAARGOS_1055 is shown as the outermost track in gold. The most inner two rings in black show the location of coding genes on the reverse and forward strands. The following rings (from inner to outer) show the conservation of homologs of the gene in *C. kefirresidentii* (orange), *C. tuberculostearicum* (purple), and *C. aurimucosum* type E (blue) genomes. The colocated set of genes found to be conserved in 80% of *C. kefirresidentii* featuring five molybdopterin associated enzymes is highlighted in gray and zoomed in at 10×.

Because prior taxonomic classifications of genomes were incongruent with their phylogenomic placement, it was previously impossible to accurately associate individual species within the *C. tuberculostearicum* species complex to skin-related diseases. With our resolved view of the species complex, we reinvestigated the metagenomic data set from Byrd et al. (22) to assess the relative abundance of *C. tuberculostearicum*, *C. kefirresidentii*, and type E *C. aurimucosum* at different stages of atopic dermatitis for a pediatric cohort. Although *C. kefirresidentii* was commonly detected in the skin metagenomes of both subjects with atopic dermatitis and the healthy controls (Fig. 3A), the species exhibited a substantial increase in relative abundance during flares in those with atopic dermatitis (Fig. 3B; Fig. S4). No increase in relative abundance during atopic dermatitis flares was observed for *C. tuberculostearicum* or *C. aurimucosum* type E. Paired testing further showed a significant decrease in the relative abundance of *C. kefirresidentii* within subjects between flare and postflare metagenomic surveys (*P* = 6.3e-3; Fig. 3C).

To further affirm the association of *C. kefirresidentii* with atopic dermatitis flares, we applied an alignment-based approach in which metagenomic reads were mapped to single-copy core genes of the *C. tuberculostearicum* species complex and coverage of species-specific alleles was measured. *C. kefirresidentii* was more evidently present in metagenomes from flares and postflare samplings, which also tended to have the lowest viable sequencing data after filtering metagenomes for adapter sequences, quality, and human contamination (Fig. S5). We further developed a statistic to assess the abundance of *C. kefirresidentii* relative to *C. tuberculostearicum* and type E *C. aurimucosum* for subjects during flares and postflares. A decrease in coverage at *C. kefirresidentii* specific-sites relative to coverage at *C. tuberculostearicum* and type E *C. aurimucosum* specific-sites was observed for 10 of 11 subjects between paired flare and postflare metagenomic surveys (*P* = 0.0092; Fig. 3D).

To understand what potential genetic factors might be contributing to the unique association of *C. kefirresidentii* with atopic dermatitis flares, we performed comparative genomics to identify traits which were lost or gained in their genomes relative to the closely related *C. tubercuostearicum* and type E *C. aurimucosum* species. We found a total of 97 homolog groups were either enriched or depleted in *C. kefirresidentii* relative to *C. tubercuostearicum* and type E *C. aurimucosum* (Table S5). *C. tubercuostearicum* and type E *C. aurimucosum* feature a core thermonuclease that is homologous to the virulence factor *nuc* (25) from Staphylococcus aureus, but missing in all *C. kefirresidentii* genomes. In contrast, two distinct colocated sets of genes, one predicted to encode for thiamine biosynthesis and the other encoding molybdopterin-binding enzymes, were enriched in *C. kefirresidenttii* but missing or significantly lacking in *C. tubercuostearicum* and type E *C. aurimucosum* genomes (Fig. 3E).

## DISCUSSION

The *C. tuberculostearicum* species complex features the three major species of *Corynebacterium* found on human skin (11). Despite this, fewer than 30 representative genomes across all species belonging to the complex are present in the most recent GTDB release and eight of the 26 genomes (30.8%) used in this study were MAGs. The lack of genomes for the species complex, despite its prevalence on healthy skin, might be in part due to specialized culture conditions required for its growth. For instance, we recently found that a single *C. kefirresidentii* isolate grows optimally when high concentrations of compounds found in both sebum and sweat are available (9).

Here, we determine that the *C. tuberculostearicum* species complex appears to miss or encode several genes that distinguish them from other *Corynebacterium* species. Because the majority of these lack any functional annotation, future molecular characterization will be essential to further our understanding of their biological roles. Interestingly, one of these genes, core to the species yet present in less than 4% of other *Corynebacterium* species, encodes for a large collagen-like peptide. Collagen-like proteins in bacteria have previously been shown to function as adhesins of pathogens to host cells and contribute to virulence (26–29).

One species in the *C. tuberculostearicum* species complex, *C. kefirresidentii*, was further found in greater abundance in metagenomes sampled during atopic dermatitis flares. Comparative genomics between *C. kefirresidentii* with *C. tuberculostearicum* and *C. aurimucosum* type E genomes show that multiple genes differentiate *C. kefirresidentii* from its two neighboring species. Most striking are a set of five colocated molybdopterin-associated enzymes, which are commonly associated with virulence (30, 31).

Two ATCC isolates (ATCC-35693/2628 LB and ATCC-35694/FPSA) (32), designated as *C. tuberculostearicum* and originating from the original study first describing the species taken from leprosy patients (1), have more recently been used in studies to associate the species to rhinosinusitis (6) and elicitation of a specific host inflammatory signaling pathway (5). To our knowledge, these isolates lack genomes however and might currently be misclassified since other species of *Corynebacterium* have been reported to similarly produce tuberculostearic acid, the trait initially used to differentiate the species from others in the genus (7, 12, 33). Identifying genetic markers to distinguish species within the *C. tuberculostearicum* species complex and continued efforts in whole-genome and metagenomic sequencing will thus be critical to correctly associate the contributions of individual species within the complex to diseases.

## MATERIALS AND METHODS

### Genome selection and taxonomic reclassification.

We had previously identified a set of 22 genomes through an iterative process in which we first identified the clade using phylogenomics as consisting of the five GTDB defined species: *C. tuberculostearicum*, *C. tuberculostearicum*_C, *C. aurimucosum*_E, and *C. kefirresidentii* (using release R202) (11). Five additional genomes belonging to the clade were identified in NCBI which were missing in GTDB release R202 and validated to belong to one of the five species through GTDB-tk classification (18). Because genomes within the clade were found to have an ANI of at least 88% to each other, we additionally included three recently published MAGs from skin microbiomes, proposed as novel species of *Corynebacterium* (based on GTDB release 88) (14), that also exhibited a >88% ANI to one of the prior defined 22 genomes. Finally, we also included the genome for a *C. kefirresidentii* strain (LK1134) isolated in our lab from healthy skin and profiled for sebum and sweat preferences (9).

Systematic reclassification of the total set of 26 genomes proposed to belong to the *C. tuberculostearicum* species complex was performed using GTDB-tk2 (34) with the latest GTDB release R207. All genomes were assigned to one of the five previously mentioned GTDB species, except for the MAGs *C.* spp. SMGC_38 and *C.* spp. SMGC_337 (14), which appear to still correspond to novel species closely related to *C. kefirresidentii*. Because MAGs were included, the contamination and completeness of genomes was assessed using CheckM (v1.2.1) (Table S1).

For comparative genomics of the *C. tuberculostearicum* species complex against other species from the *Corynebacterium* genus, all 1,296 genomes in the genus from GTDB R207 were downloaded (10). Genome dereplication was performed using dRep (23) with a secondary ANI filter of 95% using fastANI (19). This retained a set of 187 distinct genomes, including four representatives of the *C. tuberculostearicum* species complex.

### Homolog group inference and functional annotation.

Gene calling was performed on genomes using prodigal (35) and OrthoFinder (v2.5.4) (16) was subsequently used to perform homolog group detection independently among the set of 26 *C. tuberculostearicum* species complex genomes and the genus-wide set of 187 representative *Corynebacterium* genomes. For both analyses, the course set of initial orthogroups following Markov clustering were used instead of phylogenetically-determined hierarchical orthogroups.

Functional annotation was only performed for homolog groups which comparative genomics investigations suggested were significantly more prevalent or lacking within certain phylogenetic clades. Annotation was primarily performed using individual protein sequences from such homolog groups via the EggNOG mapper webserver run with default settings (36). Consolidation of annotations was performed across proteins belonging to each homolog group. For homolog groups predicted to be enriched or depleted in the *C. tuberculostearicum* species complex relative to other *Corynebacterium*, we further attempted annotation by generating consensus amino acid sequences for each homolog group and using NCBI’s conserved domain search (37) as well as Phyre2 to assess structural similarity to structurally characterized proteins (38).

**(i) Investigation of FabG homologs in *Corynebacterium*.** Homolog group *OG0000019* was identified to feature the FabG conserved domain (37), and thus, further investigated for reliable orthology to the established type II fatty acid synthase (FAS-II) FabG1 from Mycobacterium tuberculosis H37Rv because prior reports suggest *Corynebacterium* lack FAS-II (39). Briefly, the FabG1 protein sequence from M. tuberculosis H37Rv was used as a query to search all proteins from the 187 representative *Corynebacterium* using BLASTp with an E-value threshold of 1e-5. Five different homolog groups, as delineated by OrthoFinder, were identified to feature sequences homologous to FabG1. The comprehensive set of sequences from all five homolog groups, along with select representative proteins from M. tuberculosis, was aligned using MUSCLE (40) and an approximate-maximum-likelihood phylogeny was constructed using FastTree2 (41) (Fig. S3A). While sequences were interspersed for some of the homolog groups, a discrete clade consisting of all sequences belonging to the homolog group determined to be depleted in the *C. tuberculostearicum* species complex was identified and found to recapitulate the signal of depletion (Fig. S3B). This homolog group had a median copy count of one instead of three for the coarser homolog group and featured the M. tuberculosis proteins Rv0484c, a short-chain type oxidoreductase, but not *fabG1* or any *fabG*-homologs from M. tuberculosis.

### Phylogenomics of the *C. tuberculostearicum* species complex and the representative selection of *Corynebacterium*.

We identified 1,250 homolog groups as single-copy orthologs between the 26 *C. tuberculostearicum* species complex genomes. Protein alignments were constructed using MUSCLE (v5) (40) and converted to codon alignments using PAL2NAL (42). RAxML (v8.2.12) was then used to construct an initial maximum likelihood phylogeny on a concatenation of codon alignments (1,239,366 bp) with the GTRCAT model and 1,000 bootstraps. Core codon alignments were projected onto and ordered according to the reference genome *C. kefirresidentii* FDAARGOS_1055 and, together with the RAxML phylogeny, used as input for ClonalFrameML (v1.12) (17) to infer sites affected by recombination by first using a simple model, with parameter emsim set to 100 and kappa (the relative rate of transitions to transversions) set to 5.09664. For reference projection, gaps between single-copy orthologs were set to be the length of distances in between corresponding genes in the reference genome and codon alignments were reverse complemented if the gene was predicted to occur on the antistrand. Noncore sites were specified as the gaps placed in between single copy core orthologs to appropriately model the strength of linkage between them. Results from running ClonalFrameML with a simple model were used to initialize values for R/theta, 1/delta, and nu (set to 0.126997, 0.0027686, and 0.0583416, respectively) and run ClonalFrameML with a per-branch model with the embranch_dispersion parameter set 0.1, to allow for greater dispersion in parameters among branches. The initial alignment was filtered for sites predicted to be affected by recombination and subsequently subset for 15,936 sites which lacked ambiguity for any of the 26 genomes. This alignment was finally used to construct a high-resolution, maximum likelihood phylogeny of the species complex using RAxML with the same modeling strategy as applied initially.

For phylogeny construction of the full genus, we used GToTree (43) (v1.6.36) with the set of single-copy gene sets specific to Actinomycetota (formerly Actinobaceria) and FastTree2 phylogeny inference (41).

### Statistical testing for enrichment or depletion of homolog groups in clades for comparative genomics analyses.

Assessment of enrichment or depletion of homolog groups for two sets of genomes was carried out using a two-sided permutation test based on mean copy-count differences with 100,000 resamples. This allowed us to detect homolog groups which were not merely differentially present, but also those which had greater copy-counts in one set of genomes relative to the other. A generalizable program for this testing is provided in the GitHub repository: https://github.com/Kalan-Lab/Salamzade_etal_CtuberCompGen. Multiple testing correction was performed for both comparative genomics analyses separately using the Benjamini-Hochberg procedure for *P* value adjustment to control the false discovery rate. For homolog groups found to be enriched in the four *C. tuberculostearicum* species complex representative genomes relative to the complementary set of 183 *Corynebacterium* genomes, we further required that they map to homolog groups deemed to be ≥95% near-core among the 26 *C. tuberculostearicum* species complex genomes (there was a total of 1,871 near-core homolog groups).

### Metagenomic detection of *Corynebacterium* strains and species.

Three independent sets of metagenomes profiling the microbiome of healthy skin and skin affected by atopic dermatitis were processed using a pipeline previously described (4). Briefly, metagenomes were processed for adapters, quality, and human contamination using fastp (44) and KneadData (https://github.com/biobakery/kneaddata). Mock and negative-control metagenomes from the Swaney et al. study were excluded (4) as were 22 metagenomes from the Oh et al. data set marked as “human depletion” or “whole-genome amplification” under “Notes (excluded for comparisons)” in their spreadsheet describing all samples (3). Deeply sequenced metagenomes from Oh et al. (3) were randomly downsampled to 25 million read pairs using seqtk (https://github.com/lh3/seqtk). In addition, as described previously (4), five metagenomes from the Byrd et al. data set (22) (three from subject AD01 and two from subject AD10; all five were sampled during flares) were excluded because KneadData was unable to successfully process them and failure occurred at the tandem-repeat removal stage in the pipeline (all five had low sequencing read counts; <30,000 read pairs after quality trimming). Metagenomes corresponding to bilateral samplings from the same body site from the Oh et al. and Byrd et al. metagenomes were considered individually.

Detection of strains or representative genomes was performed using StrainGST (21) with a custom database constructed from complete *Corynebacterium* genomes from NCBI, as described previously (11). Briefly, this database featured 164 distinct representative genomes following dereplication using programs for StrainGST (0.1 + 191.g7fcfbcd.dirty) database creation (21). These genomes were run through GTDB-tk with database release R207 (10, 18) to generate consistent taxonomic classifications. One genome regarded as *Corynebacterium* in RefSeq (45) at the time of database creation was classified as not part of the genus by GTDB-tk and not observed in any of the metagenomic data sets. Another genome was classified as a novel *Corynebacterium* spp. not yet given an identifier in GTDB R207. We counted the number of distinct species detected in metagenomes from the three data sets and illustrated them as a multibar graph on the phylogeny of 187 *Corynebacterium* by species name. Because *C. tuberculostearicum* and *C. tuberculostearicum* type C classified genomes were found in this study to phylogenetically group as one, we considered them as a single species and mapping of detection counts for the *C. tuberculostearicum* representative genome in StrainGST was allowed to the *C. tuberculostearicum* type C representative genome in the set of 187 genomes used for phylogeny construction and comparative genomics. The species *C. sp900539985* from the *C. tuberculostearicum* species complex, which was represented in the phylogeny of 187 genomes, was not represented in the StrainGST database as it lacks a complete genome currently. Further, there was a single representative strain/genome for each of the three species from the complex represented in the StrainGST database: *C. tuberculostearicum*, *C. aurimucosum_E*, and *C. kefirresidentii*. The relative abundance estimation for these three species was thus computed based on the relative abundance computed for the corresponding representative strains/genomes by StrainGST (*rapct*) with version 1.3.3 of the software, to incorporate updates to how relative abundance was being computed relative to earlier versions.

Benchmarking of StrainGST’s ability to detect strains not represented in the database was performed by simulating paired read files from assemblies of *Corynebacterium* genomes in GTDB release R207 which belong to species represented in the StrainGST database but do not themselves correspond to reference genomes used to construct the database using ART (Table S3) (46). These read sets thus correspond to within-species strain diversity and can allow us to check StrainGST’s sensitivity in being able to detect them. Only 10 assemblies were selected for species with more than 10 assemblies available for the analysis. We additionally created 50 mock metagenomic data sets by randomly selecting and concatenating five of the simulated read sets.

Species presence for a subject at a specific body-site or a specific stage of atopic dermatitis of a subject was determined if at least one individual subcategorized metagenomes was determined to feature the species. Similarly, average abundances for species were computed across StrianGST abundance estimates for individual metagenomes, with all subcategorized metagenomes considered, including those where StrainGST did not determine the species in question as present (abundance of 0).

**(i) Confirmation of StrainGST results using an independent approach.** StrainGST is a k-mer based approach for assessing strain presence in metagenomes. To complement it, we used an independent and complementary alignment-based approach to further gauge whether *C. kefirresidentii* were enriched during atopic dermatitis flares in the Byrd et al. (22) metagenomes. For this approach, we aligned metagenomic reads individually to 4,514 representative genes from 367 homolog groups which comparative genomics had revealed were single-copy orthologs and featured at least 20 sites where *C. kefirresidentii* had a core allele that was not observed in the complementary set of genomes in the *C. tuberculostearicum* species complex. Reads were aligned as unpaired using Bowtie 2 (47) with parameters “–very-sensitive-local –no-unal -a -x.” Reads were aligned as unpaired because gene sequences are short and read pairs can thus align poorly. Additionally, all alignments for each read are requested because multiple representative genes were considered per homolog group. We then processed alignments to each representative gene for coverage at 1× was observed for 90% of the sites. Following this preliminary scan for checking that genes were loosely covered, we assessed alignments to the gene met either of the following two criteria: (i) ≥99% identity with length ≥60 bp or (ii) ≥95% identity with length ≥100 bp for the core alignment region (excluding flanking regions to account for alignments hanging off representative gene edges). We also allowed a maximum of five small indel sites within the core alignment region for the alignment to be considered. Alignments which met these criteria were then further processed and retained if they had the best mapping score observed for a particular read (in the case of ties, all alignments with the best Bowtie 2 mapping score were retained). Finally, allele counts were tallied for each metagenome at each site in homolog group codon alignments based on mapping of the query base to the reference/representative gene position in the codon alignments. Only positions in reads which had greater than 30 base quality were considered. Tallies were aggregated across individual metagenomes for each subject at each stage of atopic dermatitis.

Results from determining the presence of alleles at sites along homolog group codon alignments for each metagenome were processed to identify the proportion of *C. kefirresidentii* specific alleles covered by at least three reads (Fig. S5).

We additionally identified *C. tuberculostearicum* and type E *C. aurimucosum* specific alleles along the same set of 367 core homolog groups and quantified the mean coverage of species-specific alleles within each metagenome (inclusive of coverage at alleles which were uncovered). This allowed us to compute a statistic measuring the abundance of *C. kefirresidentii* relative to *C. tuberculostearicum* and type E. *C. aurimucosum*. This statistic was calculated as the mean coverage of *C. kefirresidentii* specific-sites divided by the cumulative sum of mean coverage values for *C. kefirresidentii*, *C. tuberculostearicum*, and *C. aurimucosum* type E specific-sites. A value greater than 0.5 for this statistic thus indicates that *C. kefirresidentii* are more prevalent than *C. tuberculostearicum* and type E. *C. aurimucosum* within an individual metagenome, whereas a value less than 0.5 supports the opposite. Our code for metagenomic alignment and calculation of the ratio statistic is provided in the GitHub repository: https://github.com/Kalan-Lab/Salamzade_etal_CtuberCompGen.

### Statistical testing.

To test whether StrainGST-based abundance estimation of *C. kefirresidentii* was greater during flares relative to postflares for paired subjects, a one-sided paired Wilcoxon test was used. Similarly, to test if the alignment-based statistic measuring the abundance of *C. kefirresidentii* relative to *C. tuberculostearicum* and *C. aurmicosum* type E decreases between paired flare and postflare metagenomes a one-sided paired Wilcoxon test was used.

### Visualizations.

iTol was used for illustrating phylogenetic trees and associated features (48). The R library ggplot2 (49) was used to visualize heatmaps, scatterplots, and bar graphs. Circleator (50) was used to generate an overview of conservation and showcase genes highlighted by comparative genomics along the chromosome of *C. kefirresidentii* FDAARGOS_1055. BioRender was used to illustrate human anatomy illustrations.
