# Supplementary material for: Comparative Genomic and Metagenomic Investigations of the Corynebacterium tuberculostearicum Species Complex Reveals Potential Mechanisms Underlying Associations To Skin Health and Disease
Source: Microbiol Spectr. 2022 Dec 21;11(1):e03578-22. doi: 10.1128/spectrum.03578-22 (PMC9927478; doi:10.1128/spectrum.03578-22)
Supplement: Supplemental file 1 — Fig. S1 to S5. Download spectrum.03578-22-s0001.pdf, PDF file, 4.2 MB [file spectrum.03578-22-s0001.pdf]

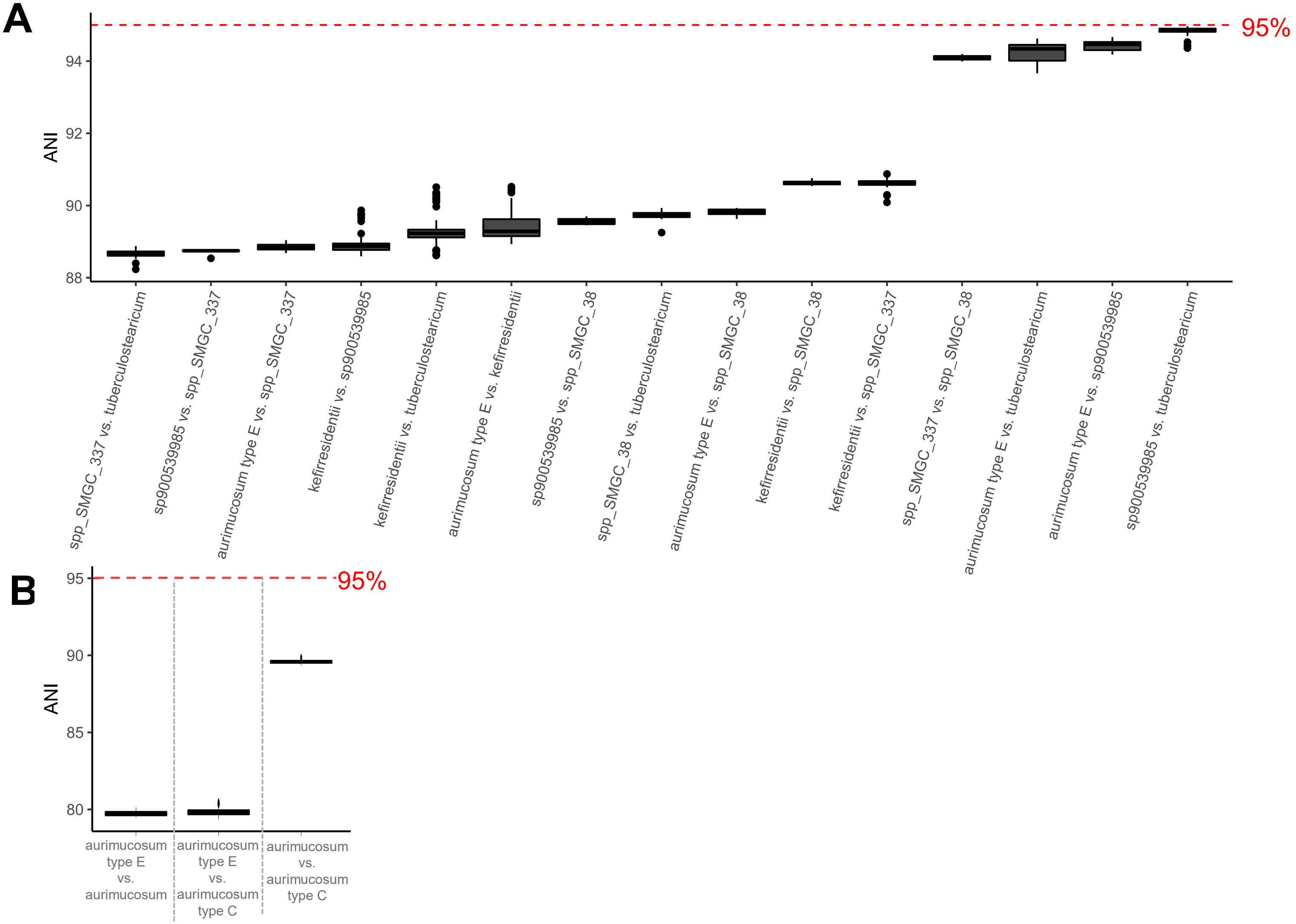

Figure S1: ANI estimates between species in the *C. tuberculostearicum* species complex. A) fastANI was used to estimate ANI between the four species within the *C. tuberculostearicum* species complex and the MAGs SMGC38 and SMGC337, which are predicted to represent closely related novel species not represented in GTDB R207. **B)** Genomes belonging to *C. aurimucosum* type E are highly divergent from genomes classified as *C. aurimucosum* and *C. aurimucosum* type C in GTDB.

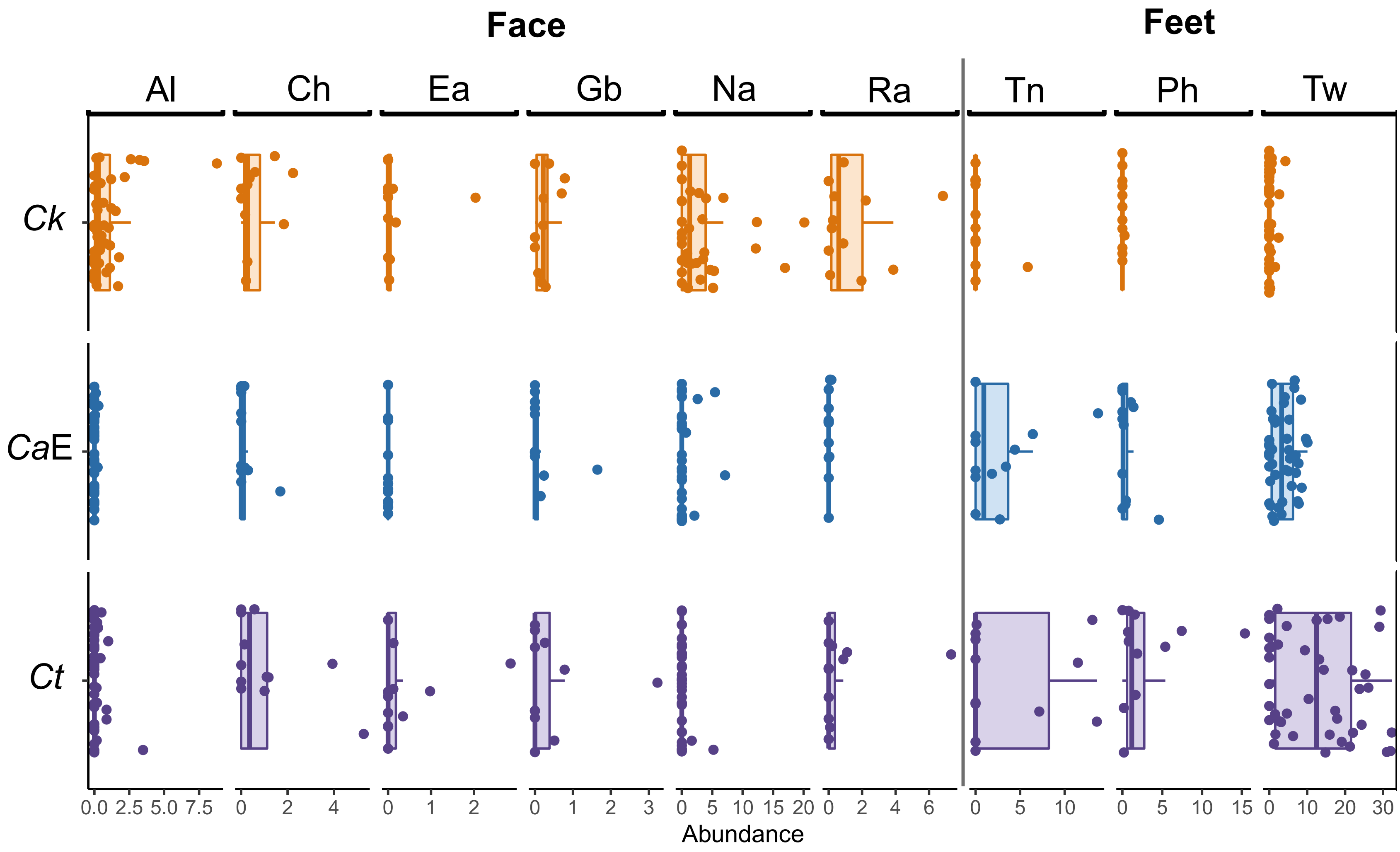

Figure S2: Abundance of *Corynebacterium tuberuclostearicum* species complex species at body sites on the face and feet. The average abundances of *C. kefirresidentii* (*Ck*), *C. aurimucosum* type E (*Ca*E), and *C. tuberculostearicum* (*Ct*) for subjects from the Oh et al. 2016 and Swaney et al. 2022 studies are shown at different body sites on the face and feet (Al = Alar crease, Ch = Cheeks, Ea = External auditory canal, Gb = Glabella, Na = Nares, Ra = Retroauricular crease, Tn = Toenail, Ph = Plantar heel, Tw = Toe-web space)

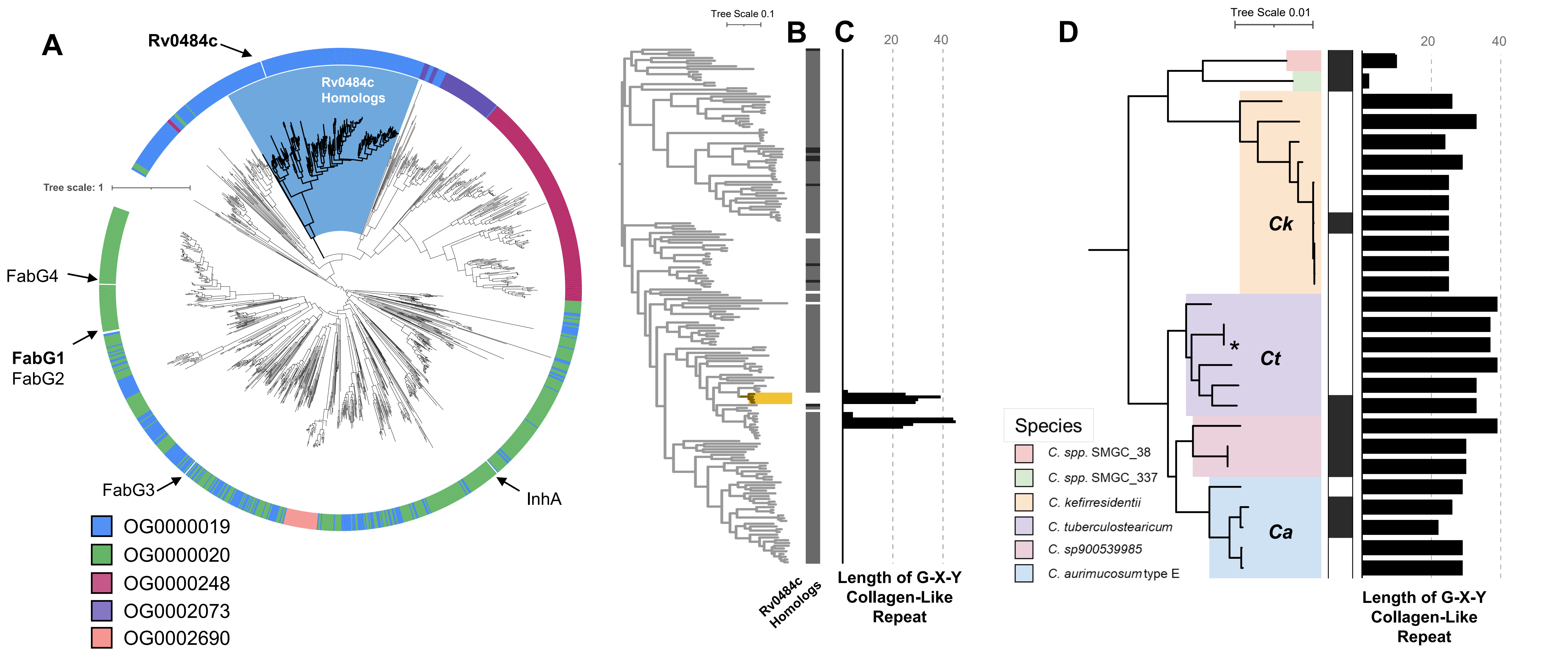

Figure S3: Investigation of depleted and enriched traits in the *C. tuberculostearicum* species complex relative to other *Corynebacterium*. A) A phylogeny of *Corynebacterium* sequences belonging to one of five homolog groups demonstrating sequence similarity to FabG1 from *M. tuberculosis*. The color strip on the outside of the phylogeny corresponds to the homolog group the sequence was designated to. The OG0000019 homolog group (blue) was found by statistical analysis to be depleted in the *C. tuberculostearicum* species complex (*Ctsc*) relative to other *Corynebacterium* species. A specific allelic clade of sequences belonging to this homolog group (highlighted as the blue clade) were found to be the origin of this signal of depletion in the *Ctsc* and found to be orthologous to Rv0484c from *M. tuberculosis*, a short-chain reductase. **B**) The more resolute allelic clade of sequences highlighted in (**A**) is depleted in the *Ctsc* but largely found in single copy in other *Corynebacterium* species. Light grey denotes a single copy of the protein is present in a genome whereas dark grey corresponds to multiple-copies in a genome. **C**) The presence of a collagen-like peptide that was found to be enriched in *Ctsc* relative to other *Corynebacterium* is shown as a bargraph with the length of bars corresponding to the number of G-X-Y motifs found in the peptide for a particular genome. **D**) The number of G-X-Y motifs found in the collagen-like peptide for genomes in the *Ctsc* is shown. The * denotes a single genome in which two collagen-like peptides mapping to the relevant homolog group were detected (likely due to early truncation as the genes are adjacent to one another).

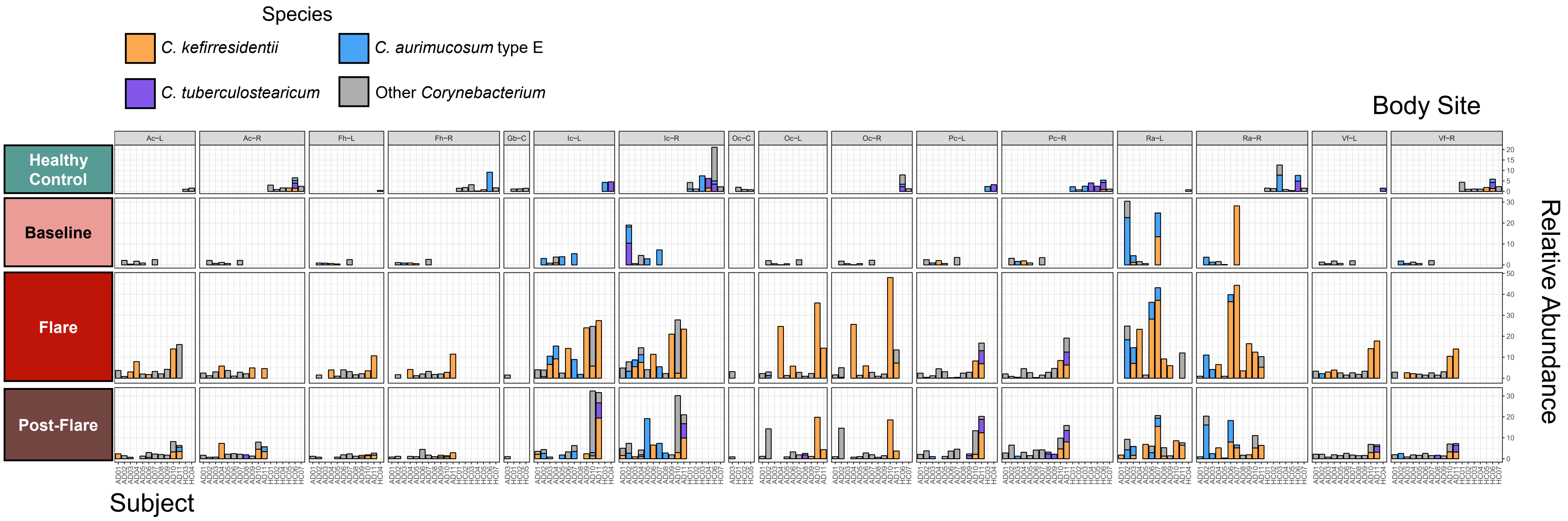

Figure S4: Increase in *C. kefirresidentii* relative abundance is observed at multiple body sites during flares. The relative abundance of *C. tuberculostearicum*, *C. aurimucosum* type E, *C. kefirresidentii,* and other *Corynebacterium* represented in the StrainGST database is shown for healthy control, baseline, flare, post-flare metagenomes from Byrd *et al.* 2017. Vertical panels correspond to body-sites (Ac = antecubital crease, Fh = forehead, Ic = inguinal crease, Oc = occiput, Pc = popliteal crease, Ra = retroauricular crease, Vf = volar forearm), with –R and –L corresponding to sampling taken from the right and left sides of the corresponding body site, respectively.

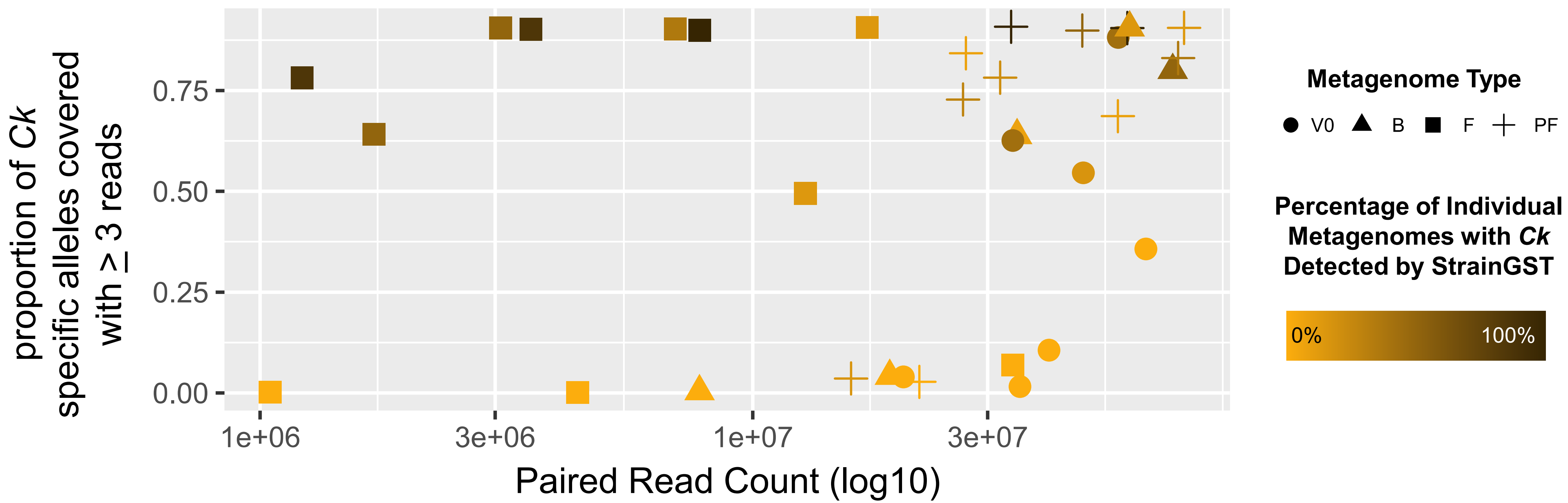

Figure S5: Independent read-alignment approach supports StrainGST detection of *C. kefirresidentii*. The figure illustrates the relationship of sequencing depth and proportion of *C. kefirresidentii* (*Ck*) specific alleles along the *C. tuberculostearicum* species core which are covered by at least 3 reads for each subject–AD category/stage pairing. Sequencing depth and individual allelic depth were aggregated across body-sites and replicate metagenomes for each pairing. The shape of dots corresponds to the AD category/stage (V0=healthy control, B=baseline, F=flare, and PF=post-flare) and the coloring corresponding to the percentage of individual metagenomes belonging to the subject-AD category/stage pairing in which StrainGST detected *C. kefirresidentii* as present.
